# Efficacy and Safety of Celecoxib Therapy in Osteoarthritis: A Meta-Analysis of Randomized Controlled Trials

**DOI:** 10.1097/MD.0000000000003585

**Published:** 2016-05-20

**Authors:** Chao Xu, Ke Gu, Yalikun Yasen, Yanjie Hou

**Affiliations:** From the Department of Orthopaedics, the Second Affiliated Hospital of Xinjiang Medical University, Urumchi (CX, YY, YH); and Department of Pain and Minimally Invasive, the 316th Hospital of People's Liberation Army, Beijing (KG), China.

## Abstract

Osteoarthritis (OA) is the most common form of arthritis in older individuals and is among the most prevalent and disabling chronic conditions worldwide.

We conducted a meta-analysis to determine the efficacy and safety of celecoxib, a cyclooxygenase-2 (COX-2) inhibitor in the treatment of osteoarthritis. Studies were pooled, and mean difference (MD), relative risk (RR), and its corresponding 95% confidence interval (CI) were calculated. Fifteen relevant articles were included for this meta-analysis study.

We observed that osteoarthritis total score (MD = −4.41, 95% CI −7.27 to −1.55), pain subscale score (MD = −0.86, 95% CI −1.10 to −0.62), and function subscale score (MD = −2.90, 95% CI −5.12 to −0.67) in OA patients treatment with celecoxib was significantly improved than that with placebo. There was no significant difference in the incidence of adverse events (AEs), SAEs, and discontinuations due to AEs; however, the incidence of gastrointestinal AEs in OA patients treatment with celecoxib is significantly higher than that with placebo. For AE, the incidence of abdominal pain in OA patients with celecoxib was significantly higher than that with placebo (RR = 2.24, 95% CI: 1.40–3.58; *P* = 0.839, *I*^2^ = 0%). There was no significant difference in diarrhea, dyspepsia, headache, and nausea.

This meta-analysis indicated that celecoxib treatment (200 mg orally once daily) led to significant improvement in the pain and function of osteoarthritis. However, compared with placebo control, celecoxib resulted in greater gastrointestinal AEs, especially abdominal pain after approximately 10 to 13 weeks of treatment. The current study, therefore, provides valuable information to help physicians make treatment decisions for their patients with OA.

## INTRODUCTION

Osteoarthritis (OA) is a type of joint disease, destruction and loss of articular cartilage, subchondral bone changes, and synovitis. These physical changes leading to chronic pain, stiffness, and disability, which are those suffering from this condition, a marked adverse impact on quality of life of patients.^1,2^ OA is one of the leading causes of disability in the world, with an aging population and an increase in obesity rates, and its prevalence is increasing in Asia.^3^

Pain relief is the main indication for drug treatment in patients with OA, most OA patients visit a doctor for their pain treatment.^4,5^ Clinical trial data show that the traditional nonsteroidal antiinflammatory drugs (NSAIDs) are more effective than acetaminophen in the treatment of patients with symptoms and signs of OA.^6,7^ However, NSAIDs possess significant limitations for serious adverse events (SAEs), including upper gastrointestinal (GI) ulceration and hemorrhage, that is associated with their use.^8,9^ In addition, older persons, so most of the traditional NSAID-related adverse events (AEs) are at particularly high risk for severe upper GI tract in patients with OA.^10,11^ The traditional NSAIDs are nonspecific inhibitors of the enzyme cyclooxygenase 2 isoforms (COX-1 and COX-2), prostaglandin synthesis of arachidonic acid catalyzed by 2 key steps.^12,13^ As a result, the traditional NSAIDs produce mechanism-based GI toxicity by inhibiting platelet COX-1.

The selective COX-2 inhibitor, celecoxib (Celebrex), in contrast, spares COX-1 in therapeutic and supra-therapeutic dosages^14,15^ to provide effective anti-inflammatory and analgesic effect,^16^ there is no increased risk with use of NSAIDs properties of GI and hematologic AEs, even though the long-term management.^17,18^ In order to determine the efficacy, GI safety, and tolerability of celecoxib (a COX-2 inhibitor) for the treatment of OA, we conducted this meta-analysis.

## MATERIALS AND METHODS

### Search Strategy

We are looking for relevant research to August, 2015 with the following terms and their combinations through PubMed and EMBASE databases: “celecoxib” and “osteoarthritis.” All scan summary, research, and references were reviewed. Only English-language publications include. In addition, reference is also retrieved the manuscript is manually search for further relevant publications. Ethical review is not required for the meta-analysis study.

### Selection Criteria

Controlled clinical trials to assess the efficacy and safety of celecoxib in OA were included if they meet the following criteria: eligibility is limited to randomized controlled trials of OA; study compared the efficacy and safety of celecoxib in treatment of OA pain; research report specific data related pain intensity and decrease AEs; and only placebo randomized controlled trials published in English may be included, both blinded and nonblinded trials were included.

### Data Extraction

All the available data were extracted from each study by 2 investigators independently according to the inclusion criteria listed above. The efficacy outcomes were: OA total score; OA pain subscale score; and OA function subscale score. The safety outcomes included: AEs, SAEs, GI AEs, discontinuations due to AEs, abdominal pain, diarrhea, dyspepsia, headache, and nausea.

### Statistical Analysis

All results summarized using STATA Software (version 12, StataCorp, College Station, TX). We calculated the mean difference (MD) and 95% confidence intervals (CI) for the continuous data, and calculate the risk ratio (RR) and 95% CIs for dichotomous data. Preliminary analysis using a fixed effect model (Mantel–Haenszel method), if there are study heterogeneity (*P* < 0.1), using a random effects model. By Begg funnel plot and Egger test to assess publication bias visually evaluated symmetry (*P* < 0.05 was considered statistically significant).

## RESULTS

### Characteristics of the Studies

**FIGURE 1:**
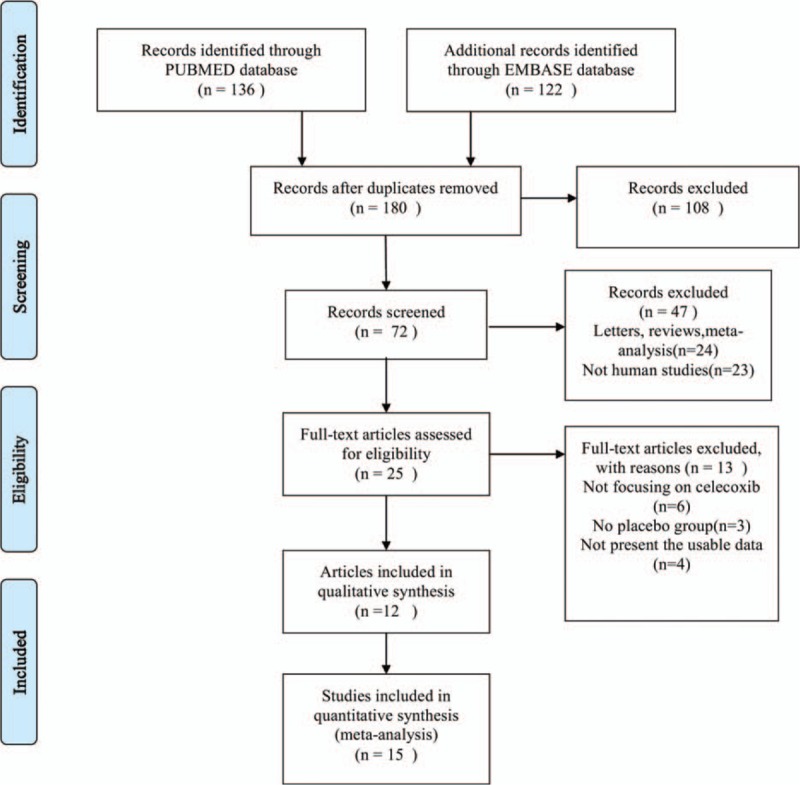
Flow diagram of studies identification.

There were 258 papers relevant to the search words. Subsequently, 233 irrelevant articles were excluded. The remaining articles were systematically reviewed, and all 25 articles qualified for full-text reading. After full-text reading, 13 articles were deemed unsuitable and were therefore excluded, and 12 articles were identified to be included for qualitative analysis. Finally, 12 articles including 15 studies^16,19–29^ were incorporated into the current meta-analysis (Table 1). The flow-chart of selection of studies and reasons for exclusion is presented in Figure 1. The pharmacokinetic and metabolic characteristics of celecoxib are summarized in (Table 2).

**TABLE 1:**
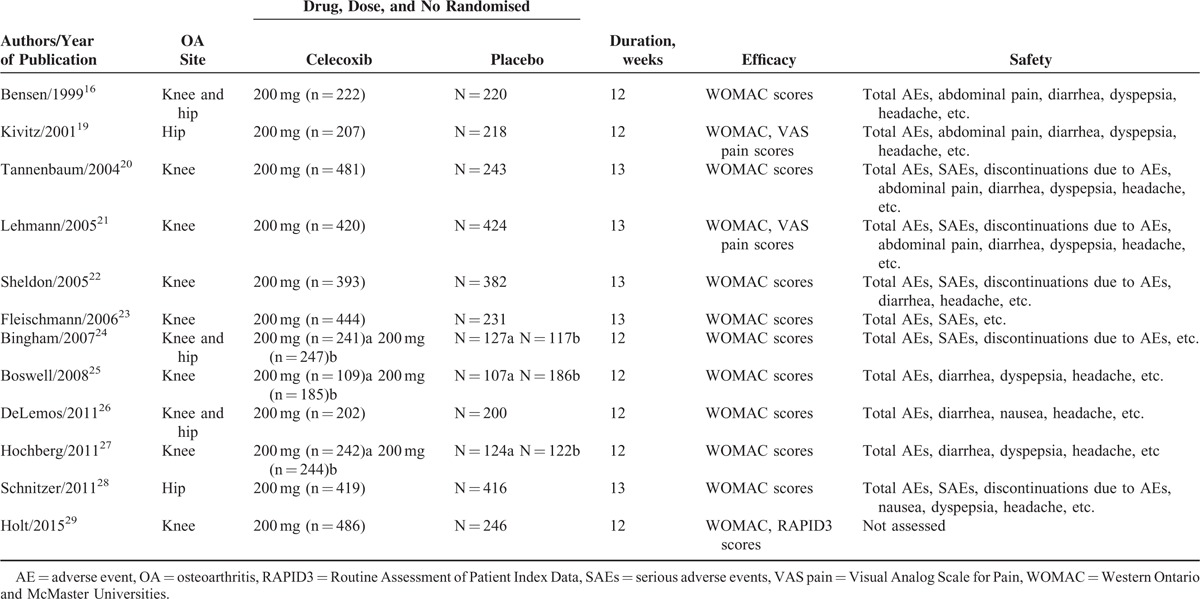
Characteristics of Randomised Controlled Trials Included in This Meta-Analysis

**TABLE 2:**
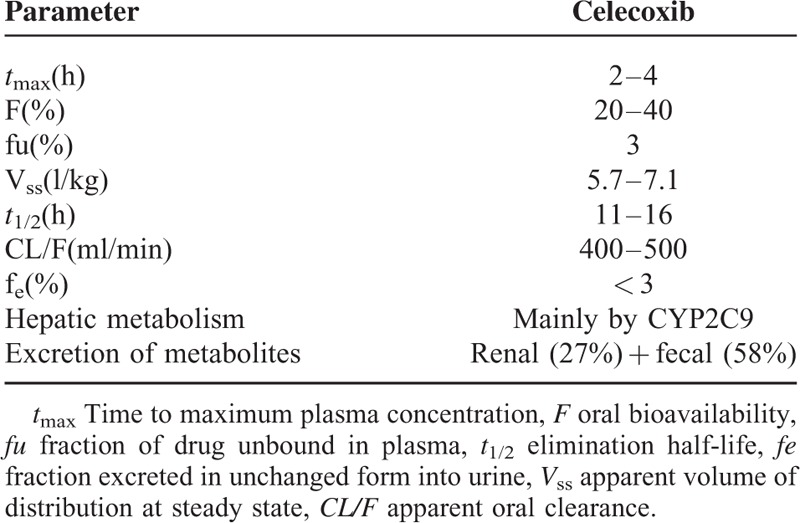
Pharmacokinetic characteristics of Celecoxib^30^

### Quantitative Synthesis

All 15 studies including 7868 patients explored the efficacy and safety of celecoxib for the treatment of OA patients.

### Osteoarthritis Total Score

**FIGURE 2:**
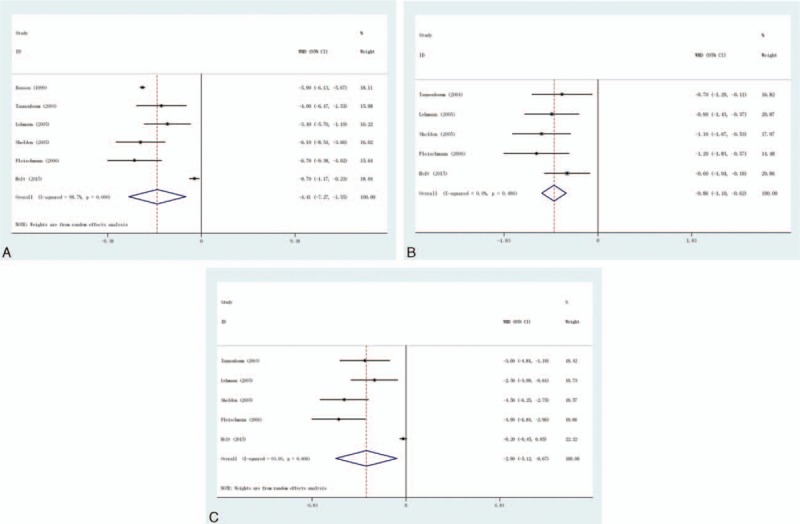
Efficacy outcomes at 10 to 13 weeks in randomised controlled trials of celecoxib versus placebo. (A) Osteoarthritis total score, (B) osteoarthritis pain subscale score, and (C) osteoarthritis function subscale score.

This outcome was reported in 6 trials, all comparing celecoxib to placebo. There were 4155 cases of patients, 2426 cases in treatment group, and 1729 cases in control group. The heterogeneity was statistically significant (*P* < 0.001, *I*^2^ = 98.7%), the random effect model was used. The difference in the OA total score was significant (MD = −4.41, 95% CI −7.27 to −1.55), as shown in Figure 2A.

### Osteoarthritis Pain Subscale Score

This outcome was reported in 5 trials, all comparing celecoxib to placebo. There were 3750 cases of patients, 2224 cases in treatment group, and 1526 cases in control group, the heterogeneity was not statistically significant, the fixed effect model was used (*P* = 0.486, *I*^2^ = 0%). The difference in the OA pain subscale score was significant (MD = −0.86, 95% CI −1.10 to −0.62), as shown in Figure 2B.

### Osteoarthritis Function Subscale Score

This outcome was reported in 5 trials, all comparing celecoxib to placebo. There were 3750 cases of patients, 2224 cases in treatment group, and 1526 cases in control group, the heterogeneity was statistically significant, the random effect model was used (*P* < 0.001, *I*^2^ = 93%). The difference in the OA function subscale score was significant (MD = −2.90, 95% CI −5.12 to −0.67), as shown in Figure 2C.

### Adverse Events (AEs)

**FIGURE 3:**
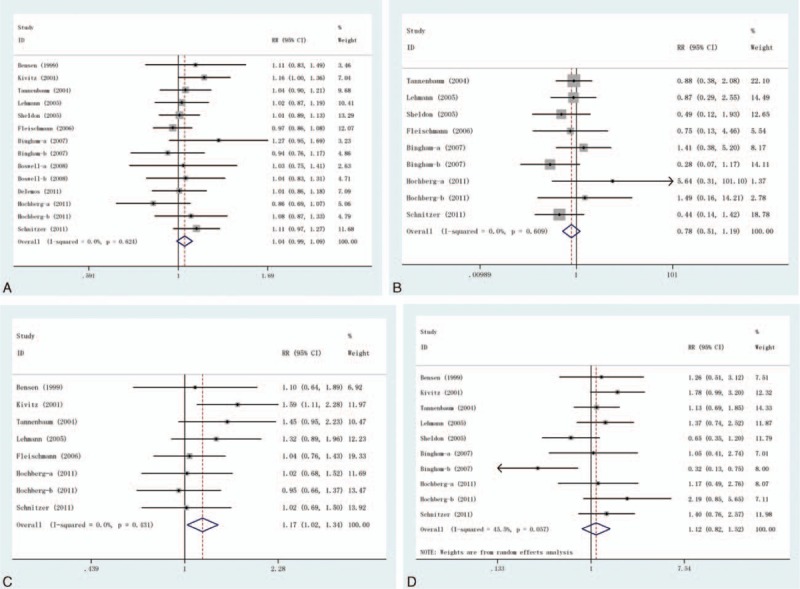
Adverse effects of treatment in randomised controlled trials of celecoxib versus placebo at 10 to 13 weeks. (A) AEs, (B) SAEs, (C)GI AEs, and (D) discontinuations due to AEs. AE = adverse event, GI = gastrointestinal, SAE = serious adverse event.

This outcome was reported in 14 trials, all comparing celecoxib to placebo. A total of 7136 patients were enrolled, 4036 patients in the treatment group and 3100 cases in the control group, there was no heterogeneity between the study (*P* = 0.624, *I*^2^ = 0%), the fixed effect model was used. There was no significant difference in the incidence of AEs (RR = 1.04, 95% CI: 0.99–1.09), as shown in Figure 3A.

### Serious Adverse Events (SAEs)

This outcome was reported in 9 trials, all comparing celecoxib to placebo. A total of 5317 patients were enrolled, 3131 patients in the treatment group and 2186 cases in the control group, there was no heterogeneity between the study (*P* = 0.609, *I*^2^ = 0%), the fixed effect model was used. There was no significant difference in the incidence of SAEs (RR = 0.78, 95% CI: 0.51–1.19), as shown in Figure 3B.

### Gastrointestinal AEs

This outcome was reported in 8 trials, all comparing celecoxib to placebo. A total of 4640 patients were enrolled, 2659 patients in the treatment group and 1981 cases in the control group, there was no heterogeneity between the study (*P* = 0.431, *I*^2^ = 0%), the fixed effect model was used. However, there was significant difference in the incidence of GI AEs (RR = 1.17, 95% CI: 1.02–1.34), as shown in Figure 3C.

### Discontinuations Due to AEs

This outcome was reported in 10 trials, all comparing celecoxib to placebo. A total of 5472 patients were enrolled, 3096 patients in the treatment group and 2376 cases in the control group, the heterogeneity was statistically significant (*P* = 0.057, *I*^2^ = 45.5%), the random effect model was used. However, there was no significant difference in the incidence of GI AEs (RR = 1.12, 95% CI: 0.82–1.52), as shown in Figure 3D.

### Abdominal Pain

**FIGURE 4:**
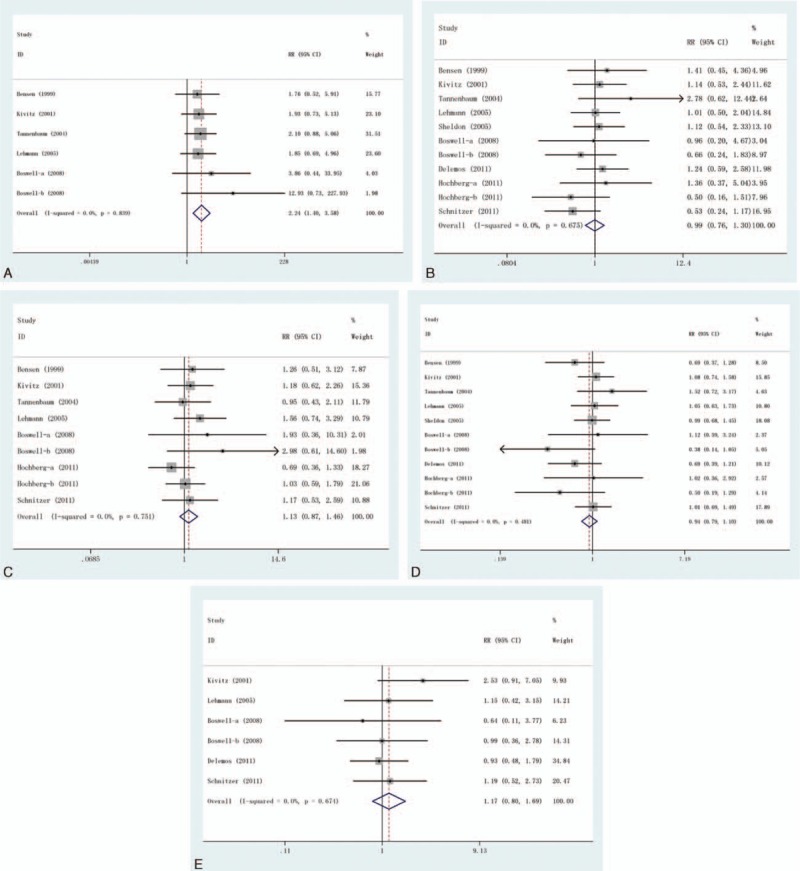
Forest plot of the RR and 95% CIs of studies on the incidence of AEs in patients with osteoarthritis treatment with celecoxib compared with placebo. (A) Abdominal pain, (B) diarrhea; (C) dyspepsia, (D) headache, and (E) nausea. AE = adverse event, CI = confidence interval, RR = relative risk.

This outcome was reported in 6 trials, all comparing celecoxib to placebo. A total of 2985 patients were enrolled, 1604 patients in the treatment group and 1381 cases in the control group, there was no heterogeneity between the study (*P* = 0.839, *I*^2^ = 0%), the fixed effect model was used. However, there was significant difference in the incidence of abdominal pain (RR = 2.24, 95% CI: 1.40–3.58), as shown in Figure 4A.

### Diarrhea

This outcome was reported in 11 trials, all comparing celecoxib to placebo. There were 5729 cases of patients, 3104 cases in treatment group and 2625 cases in control group, the heterogeneity was not statistically significant, the fixed effect model was used (*P* = 0.675, *I*^2^ = 0%). But there was no significant difference in the incidence of diarrhea (RR = 0.99, 95% CI: 0.76–1.30), as shown in Figure 4B.

### Dyspepsia

This outcome was reported in 9 trials, all comparing celecoxib to placebo. There were 4552 cases of patients, 2509 cases in treatment group and 2043 cases in control group, the heterogeneity was not statistically significant, the fixed effect model was used (*P* = 0.751, *I*^2^ = 0%). But there was no significant difference in the incidence of dyspepsia (RR = 1.13, 95% CI: 0.87–1.46), see Figure 4C.

### Headache

This outcome was reported in 11 trials, all comparing celecoxib to placebo. There were 5729 cases of patients, 3104 cases in treatment group and 2625 cases in control group, the heterogeneity was not statistically significant, the fixed effect model was used (*P* = 0.481, *I*^2^ = 0%). But the difference in the incidence of headache was not significant (RR = 0.94, 95% CI: 0.79–1.10), as shown in Figure 4D.

### Nausea

This outcome was reported in 6 trials, all comparing celecoxib to placebo. There were 3093 cases of patients, 1542 cases in treatment group and 1551 cases in control group, the heterogeneity was not statistically significant, the fixed effect model was used (*P* = 0.674, *I*^2^ = 0%). However, there was no significant difference in the incidence of nausea (RR = 1.17, 95% CI: 0.80–1.69), as shown in Figure 4E.

### Publication Bias

**FIGURE 5:**
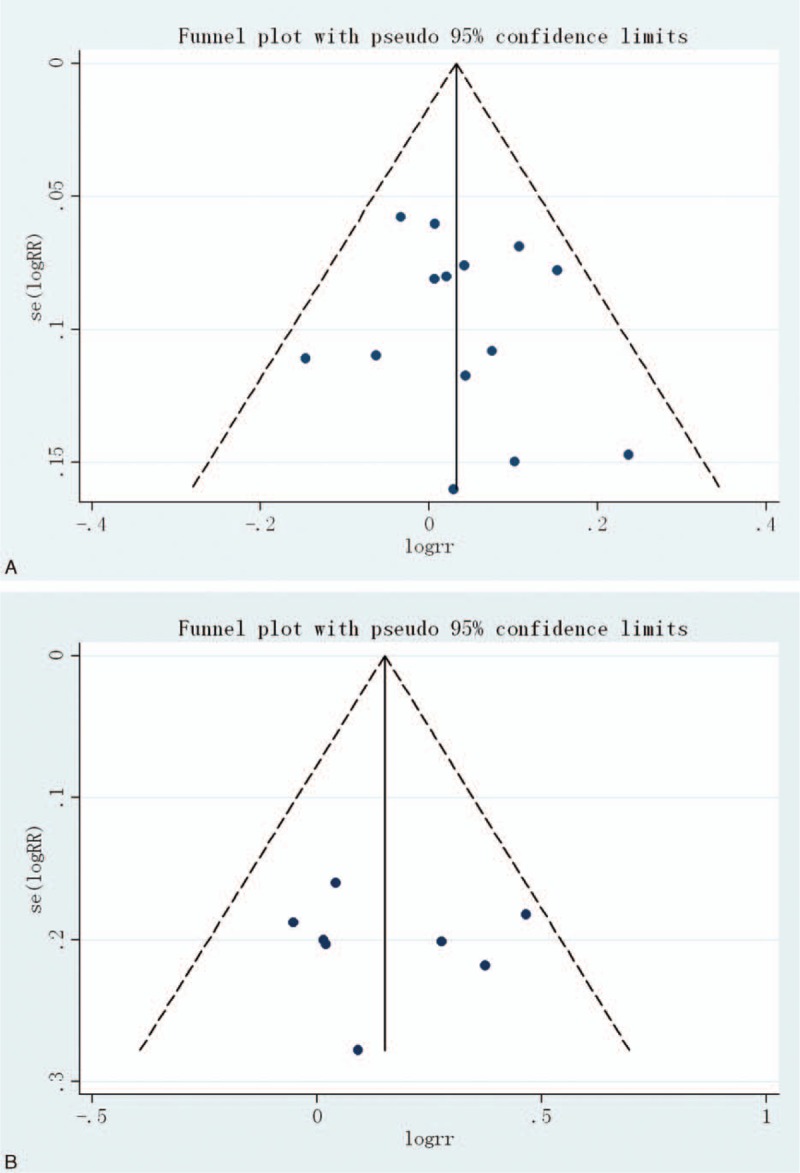
Begg funnel plot for publication bias test. Each point represents a separate study for the indicated association. (A) Incidence of adverse events, (B) incidence of GI AEs. AE = adverse event, GI = gastrointestinal.

Finally, the Egger regression test showed no evidence of asymmetrical distribution in the funnel plot in incidence of AEs (Begg test *P* = 0.661; Egger test *P* = 0.560) and incidence of GI AEs (Begg test *P* = 0.386; Egger test *P* = 0.847) (Figure 5).

## DISCUSSION

NSAIDs produce analgesic and antiinflammatory effects through inhibition of COX activity in the arachidonic acid cascade, and thereby blocking prostaglandin (PG) synthesis.^30^ In the 1990s, 2 COX isoenzymes – COX-1 and COX-2 – were identified.^31,32^ As we all know, under normal physiological circumstances, COX-1 is constitutively expressed in most tissues for participation and PG synthesis, PG protects the stomach mucosa and platelet function and maintains kidney function.^33,34^ Instead, COX-2 has been proven to be produced in inflammatory tissues and participation in the synthesis of PG correlated with inflammation.^35,36^ Therefore, conventional NSAIDs block the COX-1 and COX-2 to act a therapeutic part role, in varying degrees, through inhibition of COX-2. Side effects of NSAIDs, especially in the upper GI tract, is believed to be caused mainly by inhibition of COX-1 activity, which is involved in the protection of stomach mucosa.^37,38^ Therefore, to minimize the adverse effects, NSAIDs that target COX-2, while keeping its antiinflammatory and analgesic action. In 1998, celecoxib was approved as the 1st selective COX-2 inhibitors.^38^ Celecoxib is a member of the coxib family, which included in the anatomical therapeutic chemical classification system; it is made of a 3-dimensional structure design of molecules with COX-2 selectivity.^39^ Celecoxib is a sulfonamide and can produce severe hypersensitivity reactions in patients with sulfonamide allergies, resulting in AEs that include asthma, nasal polyps, and rhinitis.^40^

In this study, we conducted a meta-analysis to determine the efficacy and safety of celecoxib for the treatment of OA patients. Fifteen relevant studies including 7868 patients were included for this meta-analysis study. We observed that OA total score (MD = −4.41, 95% CI −7.27 to −1.55), pain subscale score (MD = −0.86, 95% CI −1.10 to −0.62), and function subscale score (MD = −2.90, 95% CI −5.12 to −0.67) in OA patients treatment with celecoxib was significantly improved than that with placebo. There was no significant difference in the incidence of AEs, SAEs, and discontinuations due to AEs; however, the incidence of GI AEs in OA patients treatment with celecoxib is significantly higher than that with placebo. For AE, the incidence of abdominal pain in OA patients with celecoxib was significantly higher than that with placebo (RR = 2.24, 95% CI: 1.40–3.58; *P* = 0.839, *I*^2^ = 0%). There was no significant difference in diarrhea, dyspepsia, headache, and nausea. The current study, therefore, provides valuable information to help physicians make treatment decisions for their patients with OA.

As already been noted, COX-2 expression in the kidney constitutively, which is adjusted in response to changes in intravascular volume.^41^ The formation of COX-2-dependent prostaglandin is necessary for normal kidney function. COX-2 inhibitors may temporarily reduce urinary sodium excretion and may cause mild to moderately elevated blood pressure.^42,43^ For kidney damage, coxibs and tNSAIDs have similar outcomes. They can cause renal insufficiency, hypertension, sodium retention and peripheral edema, hyperkalemia, and papillary necrosis.^42^ An observational study has shown that rofecoxib but not celecoxib or tNSAIDs was correlated with an increased risk of the elevation of blood pressure and edema than nonusers of any drug.^44^ Recently, in a large meta-analysis of 114 studies including 116,094 patients, rofecoxib was correlated with an increased risk of peripheral hypertension, edema, and kidney dysfunction. By contrast, celecoxib was correlated with the risk of the control group.^45^

There are several limitations in this meta-analysis that should be addressed. First, our study may be impaired to extract the raw data from including research. Second, in this study, there is potential for publication bias, because we do not take some unpublished papers and abstracts, and consider their data are not available to us. A third of a potential limitation is that language can also introduce a bias. Specifically, we select only the English language, the exclusion of other qualified researchers. Despite these limitations, this is the 1st example of a meta-analysis on the efficacy and safety of celecoxib for the treatment of OA patients. Application of statistical methods to the results of several studies with our meta-analysis, and to achieve strong objectivity, all the research methods were strict inclusion and exclusion criteria to demonstrate the effectiveness and significance of our conclusions.

In conclusion, this analysis suggests celecoxib (200 mg orally once daily), compared with placebo control, resulted in a greater reduction in pain and improved function, and acceptable adverse effects for the treatment of OA pain after approximately 10 to 13 weeks of treatment. Further studies with larger data set and well-designed models are required to validate our findings.
